# Over-Expressed Testis-Specific Protein Y-Encoded 1 as a Novel Biomarker for Male Hepatocellular Carcinoma

**DOI:** 10.1371/journal.pone.0089219

**Published:** 2014-02-20

**Authors:** Shan Li, Cuiju Mo, Shan Huang, Shi Yang, Yu Lu, Qiliu Peng, Jian Wang, Yan Deng, Xue Qin, Yinkun Liu

**Affiliations:** 1 Department of Clinical Laboratory, First Affiliated Hospital of Guangxi Medical University, Nanning, Guangxi, China; 2 Liver Cancer Institute, Zhongshan Hospital, Fudan University, Shanghai, China; 3 Cancer Research Center, Institute of Biomedical Science, Fudan University, Shanghai, China; Xiangya Hospital of Central South University, China

## Abstract

Hepatocellular carcinoma (HCC) is a male-predominant cancer. Previous studies have focused on the sex-related disparity in HCC, but the underlying mechanism remains unclear. Here, we aimed to discover characteristic biomarkers for male HCC. Clinical samples were subjected to iTRAQ labeling followed by 2DLC-ESI-MS/MS analysis. Seventy-three differential proteins containing 16 up-regulated and 57 down-regulated proteins were screened out in the male HCC group compared to that in female HCC group. Testis-specific Protein Y-encoded 1(TSPY1) is characteristically present in male HCC and was chosen for further investigation. The data from the functional effects of TSPY1 indicated that over-expression of TSPY1 could potentiate HCC cell proliferation, increase soft agar colonization, induce higher cell invasive ability and correlate with the metastatic potential of the HCC cell lines. In addition, TSPY1 and androgen receptor (AR) were co-expressed simultaneously in HCC cell lines as well as in HCC tissue. TSPY1 up- or down-regulation could lead to a high or low level expression of AR. These results implied that TSPY1 may be included in the regulation of AR expression involved in male HCC and it may act as a novel biomarker for male HCC.

## Introduction

Hepatocellular carcinoma (HCC) is one of the most common malignancies in the world, rank third cancer mortality globally [1], and there are more than 500,000 new patients with HCC worldwide every year. HCC occurs mainly in men, it is the fifth most common cancer for male and the seventh for female, the ratio of male to female is usually in the range of 2∶1 and 4∶1 [2]. In western European, the age-standardized incidence rate for male HCC was 6.2/10 million and 1.7/10 million for female, 37.9/10 million and 14.2/10 million for Chinese male and female, respectively [2]. It suggested that the difference of gender may be closely associated with the occurrence and development of HCC.

Proteomics has became a new biological research hotspot in the post-genomic era and showed an increasing important role in the biomarker discovery [3]. Proteomics technology can analyze alteration of protein molecules which play important role in forecasting for tumor development, metastatic and recurrence from the overall perspective. At present, there have been reclaimed valuable results of HCC proteomics using isobaric tag for relative and absolute quantitation (iTRAQ) technique. Previous studies observed the alteration of protein profiling in HBV-infected HepG2 cells by iTRAQ-coupled 2D LC-MS/MS technology; 15 proteins have been identified as down-regulation, including S100A6 and AnnexinA2 [4]. Chaerkady et.al found 59 up-regulated and 96 down-regulated proteins in liver cancer by comparing HCC tissues and adjacent normal tissue using the above technology [5]. But there is few proteomics research of sex-related disparity for liver cancer currently. ITRAQ is a high-throughput, reproducible and high sensitivity proteomics analysis technique, and it provides a strong technology platform for our study.

Apart from smoking and alcoholism, androgen/estrogen pathway may also be considered as a possible leading cause to sex-related disparity in HCC [6]. Findings have shown that estrogen had a protective effect, while elevated activity of the androgen axis is the major contributor for HCC [7], [8]. Epidemiology reported that HBV was the most important etiologic factor, and the incidence of male HBV-related HCC was more than that of female as a ratio of 5–7∶1 [9]. AR is a new therapeutic target for HBV-related HCC, elevated level of testosterone and the genetic polymorphism of AR were significantly correlated with the risk of HBsAg carriers suffering from HCC [10]. Wu et al [11]found specifically knocked down AR can significantly reduce hepatocarcinogenesis induced by chemical carcinogen and HBV in transgenic mouse model. AR can directly combine with HBV androgen effect element (ARE) to promote HBV RNA transcription, this reaction can stimulate hepatocarcinogenesis synergies in hepatitis B virus X protein (HBx). Previous studies have been focused on the sex-related disparity in HCC, however, the mechanism of male-predominant HCC is still unclear and there are few characteristic biomarkers for male HCC. It is urgent to discover characteristic biomarkers for male HCC and it would be valuable for guiding therapy of male HCC.

Here, we focused our efforts on the difference of expression of proteins between HBV based HCC tissue of male and female using iTRAQ-based quantitative proteomic technology. TSPY1 was screened out and further confirmed by qRT-PCR and western blot. Over-expression of TSPY1 could potentiate HCC cell proliferation, increase soft agar colonization, and strengthen cell invasive ability. It was over-expressed in male HCC tissue only and may act as a novel biomarker for male HCC.

## Materials and Methods

### Ethics Statement

Access to human tissues complied with the laws of China and the guidelines of the Ethics Committee. The Medical Ethics Committee of First Affiliated Hospital of Guangxi Medical University approved this study and all participants have given written informed consent.

### Cell Lines

The human HCC cell lines HepG2(TCHu 72), SMMC7721(TCHu 52) and Huh7(TCHu 82) which are HBV-negative cell lines were obtained from the Institute of Biochemistry and Cell Biology, Chinese Academy of Sciences, Shanghai, China (http://www.cellbank.org.cn). HCC cell lines MHCC97L, MHCC97H and HCCLM3 which are HBV-positive cell lines with the same genetic background were from Liver Cancer Institute of Fudan University (Shanghai, China) [12], [13]. All cell lines were from male HCC.

### Reagents

The iTRAQ™ Reagents Kit was from Applied Biosystems (USA). TSPY1 small hairpin (sh) RNA fragments were purchased from Genechem (Shanghai, China). Rabbit polyclonal to TSPY1 was from Abcam Company. Mouse monoclonal to Flag was purchased from Sigma. Taq polymerase purchased from TAKARA. Lipofectamine 2000 was purchased from Invitrogen. Dulbecco’s modified Eagle’s (DMEM) medium, Roswell Park Memorial Institute 1640(RPMI-1640) and fetal bovine serum (FBS) were from Sigma Group and Gibco Company.

### Tissue Samples Collection and Protein Extraction

All samples were obtained from the Department of Hepatobiliary Surgery, First Affiliated Hospital of Guangxi Medical University (Nanning, China). All the patients were from the same geographic area as the normal group with the similar genetic background and lifestyle. The clinic pathological features of the samples were listed in Table 1. All the participants were negative for antibodies against hepatitis C virus (HCV), hepatitis D virus (HDV), diabetes and hypertension. Statistical analysis showed no significant difference in age, the level of serum AFP, ALT, AST and albumin, tumor size, cirrhosis and tumor stage between the male and female HCC groups. The diagnosis of HCC was confirmed by histopathological examination, no patients had undergone radiotherapy and chemotherapy before surgery. Fresh tissues were rapidly frozen in liquid nitrogen after surgical resection, and later transferred to −80°C for preservation. After removing the blood and vessels, about 0.2 g tissue was slightly cut into pieces and 1 ml lysis buffer (20 mmol/L Tris, 7 mol/L urea, 2 mol/L thiourea, 4% CHAPS, 65 mmol/L DTT, 1 mmol/L PMSF) and grinding beads were added. The samples were shocked in multifunctional sample homogenizer (eppendorf), then centrifuged and collected supernatant. The concentration of extracted total protein was determined by the Bradford method (Bio-Rad).

**Table 1:** The clinical and pathological features of all samples.

Feature	HCC		Normal		
Gender	Male	Female		Male	Female
Number of individuals	49	28		8	14
Age (years)	48±14	52±11*		41±12	47±12
Hepatitis B surface Ag					
Positive	49	28		0	0
Negative	0	0		8	14
Serum AFP (ng/ml)	68.20(0.88–60500.00)	37.50(1.53–58344.00)*		2.52(2.16–4.63)	2.84(0.88–4.88)
Serum ALT(U/L)	36.0(14.0–294.0)	24.0(10.0–80.0)*		24.0(17.0–71.0)	16.0(6.0–24.0)
Serum AST(U/L)	35.0(14.0–215.0)	31.0(23.0–107.0)*		34.0(20.0–57.0)	24.0(17.0–32.0)
Serum albumin (g/L)	38.0(24.7–45.6)	35.8(31.2–44.2)*		37.7(34.8–43.5)	39.6(37.0–47.6)
Tumor size* ^,^ a					
≤5 cm	24	9		0	0
>5 cm	25	19		0	0
Cirrhosis* ^,^ a					
Absent	28	16		0	0
Present	21	12		0	0
Tumor stage* ^,^ a					
StageI	37	18		0	0
StageII	12	10		0	0
Metastasis	0	0		0	0

*p>0.05;

a Chi-square test.

### ITRAQ Labeling and 2DLC-ESI-MS/MS

**Figure 1:**
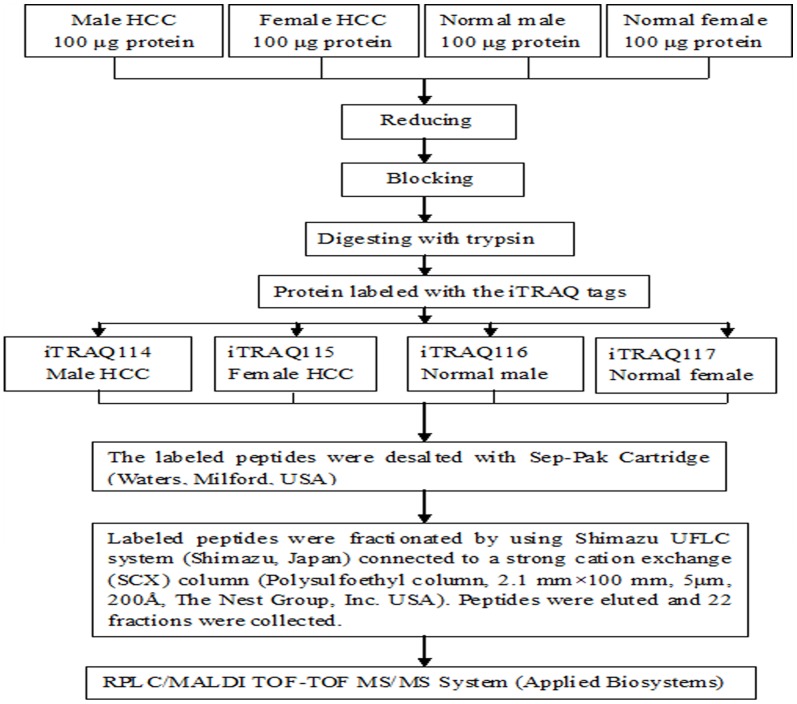
Experimental procedure of iTRAQ.

The experimental procedure of iTRAQ labeling and 2DLC-ESI-MS/MS was showed in Figure 1 according to the previous study [12]. The identification and quantification of protein for the iTRAQ were executed using ProteinPilot 3.0 software (Applied Biosystems, USA). Data searching was performed against SWISS-PROT human database according to the Paragon algorithm. At least two peptides with 95% confidence or one of the two peptides with 99% confidence were considered for protein identification. The results were exported into excel, proteins were considered as up-regulated with their ratios >1.2 and when their ratios <0.8 were considered as down-regulation [14].

The differentially expressed proteins screened by iTRAQ were further analyzed using UniProt database. The proteins were classified in Gene Ontology (GO) in accordance with biological functions and were evaluated biological process, subcellular distributions and molecular function in the SWISS-PROT database. The Reactome was used to analyze the biological pathways of the proteins.

### Lentiviral Vector Construction and Lentivirus Packaging

The CDS region of human TSPY1 was cloned from human genomic DNA and was confirmed by sequencing, the PCR product was subcloned into GV287 (Shanghai GeneChem Co., Ltd., Shanghai, China). TSPY1-shRNAs were designed to target the TSPY1 gene (NM_003308), the specific shRNA sequences (5′- TTGCTGAGATCCTATGTAA-3′ and 5′-TTAACTTCTTCAACTGGTT-3′) were cloned into the GV115 (Shanghai GeneChem Co., Ltd., Shanghai, China). A GFP-lentiviral vector was used as a negative control. The lentivirus was produced by transfecting lentiviral plasmids into HEK-293T cells with Lipofectamine 2000 (Invitrogen). After 48 h of transfection, cell supernatants containing lentivirus was harvested, concentrated and calibrated virus titer. The lentivirus with a final concentration of 8E+8 TU/ml was stored at −80°C.

### Lentiviral Transfection

MHCC97H cells and Huh7 cells were cultured in DMEM medium and SMMC7721 cell was cultured in RPMI-1640 supplemented with 10% FBS 100U/ml streptomycin, 100U/ml penicillin in a humidified atmosphere of 5% CO2 at 37°C. The day before transfection, the HCC cells were plated at a density of 1×10^5^ cells/well in six-well plates. Then the medium was replaced with 1 ml serum-free medium, and these cells were transfected with lentivirus at a multiplicity of infection (MOI) of 20 in the presence of 10 µg/ml of polybrene (Sigma). The culturing medium was changed to complete medium after 12 h of transfection. After cultured for another 72 h, the rate of infection was observed using fluorescence microscope, the total cellular protein and RNA were extracted for further validation or the transfected cells were conducted cell functional experiment.

### Quantitative RT-PCR

Total RNA was isolated from cultured cells using Trizol reagent (Invitrogen) as suggested by the manufacturer’s instructions. A total of 2 µg RNA and oligo-dT were reverse-transcribed into cDNA using a reverse transcriptase reaction kit (Ferments). RT-PCR was performed using SYBR Green PCR Master Mix and reactions (TAKARA) on IQ5 Multicolor Real-time PCR Detection System (Bio-Rad) following the conditions: 95°C for 5 min, 40 cycles of 95°C for 15 s, 59°C for 15 s and 72°C for 20 s. The experimental Ct (cycle threshold) was normalized to β-actin control product and all of the amplifications were performed in three technical repeats. The amount of target gene relative to those expressed by mock cells was calculated by 2^−ΔΔT^ method. The primer sequences were listed in Table 2.

**Table 2:** List of primers used for RT-PCR.

Gene	Primer sequences
TSPY1	Forward: 5′-ATGTTGTTCTTTCGGAGTAACCC-3′
	Reverse: 5′-TGAGAAGCCCTGTATTCTGTGAT -3′
B2L13	Forward: 5′-ATCACTGCCACCTCCCTG-3′
	Reverse: 5′-TTGCTGCTTTCACCTCTTCT-3′
CP27A	Forward: 5′-TTCGAGAAACGCATTGGC-3′
	Reverse: 5′-GGAGGAAGGTGGCATAGAGT-3′
AK1C1	Forward: 5′-AGACATTGTTCTGGTTGCCTAT-3′
	Reverse: 5′-GGGTTCGCTTGTGCTTTT-3′
AR	Forward: 5′-ACTCCAGGATGCTCTACTTCG-3′
	Reverse: 5′-AGGTGCCTCATTCGGACA-3′
CXCR4	Forward:5′-AACTTCCTATGCAAGGCAGT-3′
	Reverse:5′-TATCTGTCATCTGCCTCACT-3′
HIF-1	Forward:5′- TTACAGCAGCCAGACGATCA -3′
	Reverse:5′- CCCTGCAGTAGGTTTCTGCT -3′
β-actin	Forward: 5′- CATGTACGTTGCTATCCAGGC-3′
	Reverse: 5′-CTCCTTAATGTCACGCACGAT-3′

### Western Blot

A total of 20 µg proteins were separated by 10% SDS-PAGE and transfered to 0.2 µm polyvinylidene fluoride membrane (PVDF, Millipore, Billerica, USA) using a Bio-Rad SemiDry instrument (Bio-Rad). The membrane was blocked by 5% milk at room temperature for 1 h, then incubated with Flag (1∶1000 dilution),TSPY1 (1∶500 dilution) and GAPDH (1∶10000 dilution) at 4°C overnight. After 3 times of 15 min washing by 0.1% TBST (50 mmol/L Tris-HCl, 150 mmol/L NaCl, 0.1% v/v Tween-20, pH7.4), the membrane was further incubated with HRP-conjugated secondary antibodies (1∶10000 dilution) for 1 h at room temperature. Then washed again by 0.1% TBST for 3 times of 15 min, the protein bands were visible and semi-quantitative analysis using enhanced chemiluminescence detection (ECL; GE, Healthcare, Piscataway, NJ).

### Cell Proliferation and Cell Migration Assays

The cells (1000 cells/well) infected with lentiviral vector were dispensed in 100 µl medium into a 96-well plate for 24 h,48 h and 72 h, respectively. At the indicated time points, added 10 µl 2-(4-indophenyl)-3-(4-nitrophenyl)-5-(2,4-disulphophenyl)- 2H- tetrazolium monosodium salt (CCK8, Cell Counting kit) into the wells and incubated for 1 h, then the plate was read using an enzyme-linked immunosorbent assay plate reader at 450 nm.

Cell migration was performed using transwell chamber with 8 µm pores (Corning Costar, Cambridge, MA). The cells infected with lentiviral vector were trypsinized and resuspended in DMEM containing 3% FBS. Cells (5×10^4^) were plated in the upper chamber, and the upper chambers were inserted in a well of a 24-well plate containing 600 µl 20% FBS-DMEM. After incubation for 36 h, the non-migrating cells in the upper chamber were removed using a cotton swab and the cells that had migrated to the underside of the membrane were fixed with 4% paraformaldehyde for 30 min, stained with 10% Giemsa for 30 min. The cells in the underside of the membrane were counted under light microscopy.

### Cell Apoptosis

Prepare enough cells for incubating with the MuseTM Annexin V&Dead Cell Reagents and the cells were resuspended in 1% BSA-PBS. Added 100 µl of MuseTM Annexin V& Dead Cell reagent to a new tube and then mixed with 100 µl of cells in suspension. The cells were incubated for 20 min at room temperature in dark and counted by MuseTM Annexin V& Dead Cell software.

### Soft Agar Colonization

First, 1 ml of sterilized 0.6% low melting point agarose (Sigma) in complete medium was added to each well of 6-well plate. After the medium became solid gel at 37°C, 1 ml of 0.3% low melting point agarose in complete medium with 1000 cells was added on top of the base gel. The plate was cultured in cell incubator for 14 days. The number of clone (≧50 cells) was assessed microscopically. All experiments were performed in triplicate.

### Statistical Analysis

The statistical analyses were performed using a commercially available statistical software package (SPSS for Windows, 16.0). Quantitative variables were analyzed by Student’s t-test. The correlation was assessed by Pearson method. *P*<0.05 was considered as statistically significant.

## Results

### Identification and Relative Quantification of Tissue Proteome Based Sex-related Disparity for HCC

Under the condition of unused ProtScore>1.3 for ProteinPilot 3.0 software and removed the anti-library and redundant proteins, a total of 652 distinct proteins were identified and quantified. Compared with the normal person and accordance with parameters as test to normal ratio>1.2 (protein with up-regulation) or ratio <0.8 (protein with down-regulation), *p*<0.05 and relative standard deviation (EF) <2.5, 109 proteins including 26 up-regulated and 83 down-regulated proteins were screened out as differential proteins in the HCC patients. There were 71 differential proteins between the normal and HCC male groups (Table 3), and 38 proteins between normal and HCC female groups (Table 4). Compared to the female HCC group, 73 differential proteins including 16 up-regulations and 57 down-regulations were screened out in the male HCC group (Table 5).

**Table 3:** 71 proteins showed differences in expression levels between male groups at HCC group compared to normal group.

Accession	Genesymbol	Name	Peptides(95%)	114∶116	PVal	EF
Q01534	TSPY1	Testis-specific Y-encoded protein 1	1	87.90	0.0397	2.29
Q01105	SET	Protein SET	1	87.90	0.0494	2.29
Q15063	POSTN	Periostin	1	87.90	0.0465	2.29
P06733	ENOA	Alpha-enolase	7	10.00	0.0340	1.91
P49327	FAS	Fatty acid synthase	15	6.85	0.0109	1.64
P11021	GRP78	78 kDa glucose-regulated protein	13	5.55	0.0022	1.64
P08670	VIME	Vimentin	8	3.84	0.0010	1.92
P07355	ANXA2	Annexin A2	5	2.61	0.0201	1.91
P07237	PDIA1	Protein disulfide-isomerase	14	2.47	0.0003	1.80
P07737	PROF1	Profilin-1	3	2.17	0.0145	2.47
P35579	MYH9	Myosin-9	15	1.63	0.0313	1.49
Q04828	AK1C1	Aldo-keto reductase family 1 member C1	4	1.28	0.0136	1.67
P11586	C1TC	C-1-tetrahydrofolate synthase, cytoplasmic	4	0.61	0.0395	1.42
Q16822	PPCKM	Phosphoenolpyruvate carboxykinase [GTP], mitochondrial	8	0.54	0.0128	1.56
P30038	AL4A1	Delta-1-pyrroline-5-carboxylate dehydrogenase, mitochondrial	5	0.46	0.0336	1.36
P11498	PYC	Pyruvate carboxylase, mitochondrial	9	0.44	0.0136	1.42
P23141	EST1	Liver carboxylesterase 1	14	0.37	0.0001	1.54
P33121	ACSL1	Long-chain-fatty-acid–CoA ligase 1	3	0.37	0.0130	2.31
P54868	HMCS2	Hydroxymethylglutaryl-CoA synthase, mitochondrial	8	0.36	0.0032	1.74
Q13228	SBP1	Selenium-binding protein 1	2	0.35	0.0023	2.44
P68871	HBB	Hemoglobin subunit beta	18	0.34	0.0396	1.26
P00167	CYB5	Cytochrome b5	4	0.30	0.0007	1.54
P24752	THIL	Acetyl-CoA acetyltransferase, mitochondrial	7	0.28	0.0065	2.07
P07099	HYEP	Epoxide hydrolase 1	8	0.26	0.0408	1.91
P09110	THIK	3-ketoacyl-CoA thiolase, peroxisomal	4	0.24	0.0173	2.38
P69905	HBA	Hemoglobin subunit alpha	10	0.24	0.0328	1.43
P31327	CPSM	Carbamoyl-phosphate synthase [ammonia], mitochondrial	29	0.24	0.0000	1.43
P11509	CP2A6	Cytochrome P450 2A6	4	0.24	0.0261	1.71
P00367	DHE3	Glutamate dehydrogenase 1, mitochondrial	11	0.21	0.0001	2.33
O75891	FTHFD	10-formyltetrahydrofolate dehydrogenase	5	0.16	0.0017	1.87
P06576	ATPB	ATP synthase subunit beta, mitochondrial	12	0.15	0.0081	1.42
Q00796	DHSO	Sorbitol dehydrogenase	4	0.15	0.0371	2.23
P05062	ALDOB	Fructose-bisphosphate aldolase B	10	0.15	0.0002	2.49
P80404	GABT	4-aminobutyrate aminotransferase, mitochondrial	8	0.13	0.0089	1.77
O95954	FTCD	Formimidoyltransferase-cyclodeaminase	8	0.12	0.0251	2.00
Q9Y2Q3	GSTK1	Glutathione S-transferase kappa 1	1	0.01	0.0191	2.47
Q8N0X4	CLYBL	Citrate lyase subunit beta-like protein, mitochondrial	1	0.01	0.0175	2.44
P62333	PRS10	26S protease regulatory subunit S10B	1	0.01	0.0186	2.44
Q6UX53	MET7B	Methyltransferase-like protein 7B	1	0.01	0.0177	2.33
P04632	CPNS1	Calpain small subunit 1	1	0.01	0.0198	2.33
P84090	ERH	Enhancer of rudimentary homolog	2	0.01	0.0186	2.29
Q02318	CP27A	Cytochrome P450 27, mitochondrial	2	0.01	0.0187	2.27
Q13561	DCTN2	Dynactin subunit 2	1	0.01	0.0199	2.25
Q96EY8	MMAB	Cob(I)yrinic acid a,c-diamide adenosyltransferase, mitochondrial	1	0.01	0.0181	2.27
Q9Y2V2	CHSP1	Calcium-regulated heat stable protein 1	2	0.01	0.0180	2.27
Q9BVK6	TMED9	Transmembrane emp24 domain-containing protein 9	1	0.01	0.0181	2.29
P51687	SUOX	Sulfite oxidase, mitochondrial	1	0.01	0.0173	2.29
Q96I99	SUCB2	Succinyl-CoA ligase [GDP-forming] subunit beta, mitochondrial	1	0.01	0.0184	2.29
Q9Y5M8	SRPRB	Signal recognition particle receptor subunit beta	1	0.01	0.0168	2.29
P02743	SAMP	Serum amyloid P-component	1	0.01	0.0164	2.29
P50336	PPOX	Protoporphyrinogen oxidase	1	0.01	0.0187	2.29
O95487	SC24B	Protein transport protein Sec24B	1	0.01	0.0184	2.29
P05165	PCCA	Propionyl-CoA carboxylase alpha chain, mitochondrial	1	0.01	0.0198	2.29
P48147	PPCE	Prolyl endopeptidase	1	0.01	0.0200	2.29
Q00325	MPCP	Phosphate carrier protein, mitochondrial	1	0.01	0.0190	2.29
Q9NQR4	NIT2	Nitrilase homolog 2	2	0.01	0.0193	2.29
Q16795	NDUA9	NADH dehydrogenase [ubiquinone] 1 alpha subcomplex subunit 9, mitochondrial	1	0.01	0.0187	2.29
P84157	MXRA7	Matrix-remodeling-associated protein 7	1	0.01	0.0181	2.29
P04196	HRG	Histidine-rich glycoprotein	1	0.01	0.0179	2.29
P30712	GSTT2	Glutathione S-transferase theta-2	1	0.01	0.0191	2.29
P09211	GSTP1	Glutathione S-transferase P	1	0.01	0.0183	2.29
P23588	IF4B	Eukaryotic translation initiation factor 4B	1	0.01	0.0181	2.29
Q9Y262	IF3EI	Eukaryotic translation initiation factor 3 subunit E-interacting protein	1	0.01	0.0185	2.29
P27105	STOM	Erythrocyte band 7 integral membrane protein	1	0.01	0.0186	2.29
O94905	ERLN2	Erlin-2	1	0.01	0.0181	2.29
Q02338	BDH	D-beta-hydroxybutyrate dehydrogenase, mitochondrial	1	0.01	0.0161	2.29
Q9BXK5	B2L13	Bcl-2-like 13 protein	1	0.01	0.0178	2.29
O75964	ATP5L	ATP synthase subunit g, mitochondrial	1	0.01	0.0182	2.29
Q9UKK9	NUDT5	ADP-sugar pyrophosphatase	1	0.01	0.0192	2.29
P55263	ADK	Adenosine kinase	1	0.01	0.0188	2.29
P13798	ACPH	Acylamino-acid-releasing enzyme	1	0.01	0.0182	2.29

**Table 4:** 38 proteins showed differences in expression levels between female groups at HCC group compared to normal group.

Accession	Genesymbol	Name	Peptides(95%)	115∶117	PVal	EF
P61026	RAB10	Ras-related protein Rab-10	1	87.90	0.0492	2.29
P62942	FKB1A	Peptidyl-prolyl cis-trans isomerase FKBP1A	1	87.90	0.0471	2.29
P05093	CP17A	Cytochrome P450 17A1	1	87.90	0.0491	2.29
P53999	TCP4	Activated RNA polymerase II transcriptional coactivator p15	1	87.90	0.0413	2.29
P62917	RL8	60S ribosomal protein L8	1	87.90	0.0468	2.29
P02656	APOC3	Apolipoprotein C-III	1	74.47	0.0430	2.42
P51659	DHB4	Peroxisomal multifunctional enzyme type 2	4	20.32	0.0363	2.36
P04792	HSPB1	Heat shock protein beta-1	3	18.71	0.0311	2.86
P14625	ENPL	Endoplasmin	11	6.55	0.0028	2.13
P53396	ACLY	ATP-citrate synthase	3	2.38	0.0167	2.44
O00264	PGRC1	Membrane-associated progesterone receptor component 1	3	1.89	0.0380	1.58
O15260	SURF4	Surfeit locus protein 4	3	1.50	0.0465	2.03
Q96L21	RL10L	60S ribosomal protein L10-like	1	1.26	0.0498	2.51
P49411	EFTU	Elongation factor Tu, mitochondrial	3	1.22	0.0239	1.92
P11310	ACADM	Medium-chain specific acyl-CoA dehydrogenase, mitochondrial	1	0.47	0.0198	2.11
Q3LXA3	DHAK	Dihydroxyacetone kinase	4	0.27	0.0227	2.44
P00966	ASSY	Argininosuccinate synthase	5	0.17	0.0069	2.65
P02768	ALBU	Serum albumin	30	0.11	0.0001	1.27
P54727	RD23B	UV excision repair protein RAD23 homolog B	1	0.01	0.0189	2.78
P01860	IGHG3	Ig gamma-3 chain C region	4	0.01	0.0184	2.54
P22570	ADRO	NADPH:adrenodoxin oxidoreductase, mitochondrial	1	0.01	0.0188	2.42
A6NL28	TPM3L	Putative tropomyosin alpha-3 chain-like protein	1	0.01	0.0186	2.36
Q92530	PSMF1	Proteasome inhibitor PI31 subunit	2	0.01	0.0191	2.70
Q93099	HGD	Homogentisate 1,2-dioxygenase	1	0.01	0.0184	2.54
P20073	ANXA7	Annexin A7	1	0.01	0.0187	2.27
Q8NI22	MCFD2	Multiple coagulation factor deficiency protein 2	1	0.01	0.0181	2.27
P50991	TCPD	T-complex protein 1 subunit delta	1	0.01	0.0187	2.29
O15269	SPTC1	Serine palmitoyltransferase 1	1	0.01	0.0186	2.29
P62834	RAP1A	Ras-related protein Rap-1A	1	0.01	0.0182	2.29
Q9Y617	SERC	Phosphoserine aminotransferase	1	0.01	0.0177	2.29
Q14847	LASP1	LIM and SH3 domain protein 1	1	0.01	0.0189	2.29
O00410	IPO5	Importin-5	1	0.01	0.0185	2.29
Q9Y5Z4	HEBP2	Heme-binding protein 2	1	0.01	0.0184	2.29
Q9UIJ7	KAD3	GTP:AMP phosphotransferase mitochondrial	1	0.01	0.0182	2.29
P38117	ETFB	Electron transfer flavoprotein subunit beta	2	0.01	0.0189	2.29
P46977	STT3A	Dolichyl-diphosphooligosaccharide–protein glycosyltransferasesubunit STT3A	1	0.01	0.0200	2.29
P08572	CO4A2	Collagen alpha-2(IV) chain	1	0.01	0.0191	2.29
P14868	SYDC	Aspartyl-tRNA synthetase, cytoplasmic	1	0.01	0.0177	2.29

**Table 5:** 73 proteins showed differences in expression levels between HCC groups at male group compared to female group.

Accession	Genesymbol	Name	Peptides(95%)	114∶115	PVal	EF
Q06210	GFPT1	Glucosamine–fructose-6-phosphate aminotransferase[isomerizing] 1	1	87.90	0.0493	2.29
P14868	SYDC	Aspartyl-tRNA synthetase, cytoplasmic	1	87.90	0.0490	2.29
Q01534	TSPY1	Testis-specific Y-encoded protein 1	1	87.90	0.0397	2.29
Q00839	HNRPU	Heterogeneous nuclear ribonucleoprotein U	1	83.95	0.0494	2.27
P68371	TBB2C	Tubulin beta-2C chain	13	73.11	0.0487	2.42
O75891	FTHFD	10-formyltetrahydrofolate dehydrogenase	5	13.68	0.0021	1.89
P02768	ALBU	Serum albumin	30	8.71	0.0000	1.28
P08670	VIME	Vimentin	8	6.67	0.0143	2.33
Q99880	H2B1L	Histone H2B type 1-L	9	5.92	0.0483	2.07
P54868	HMCS2	Hydroxymethylglutaryl-CoA synthase, mitochondrial	8	3.44	0.0112	2.49
P09525	ANXA4	Annexin A4	5	2.58	0.0218	2.19
P18206	VINC	Vinculin	2	1.94	0.0148	2.03
P07737	PROF1	Profilin-1	3	1.77	0.0154	2.15
P11586	C1TC	C-1-tetrahydrofolate synthase, cytoplasmic	4	1.71	0.0273	1.42
P05023	AT1A1	Sodium/potassium-transporting ATPase subunit alpha-1	2	1.37	0.0491	2.16
Q04828	AK1C1	Aldo-keto reductase family 1 member C1	4	1.19	0.0180	1.87
P33121	ACSL1	Long-chain-fatty-acid–CoA ligase 1	3	0.69	0.0337	1.61
O15260	SURF4	Surfeit locus protein 4	3	0.69	0.0393	1.58
Q9Y6C9	MTCH2	Mitochondrial carrier homolog 2	4	0.65	0.0346	1.34
P35579	MYH9	Myosin-9	15	0.63	0.0055	1.25
P08684	CP3A4	Cytochrome P450 3A4	4	0.63	0.0251	1.34
P51659	DHB4	Peroxisomal multifunctional enzyme type 2	4	0.57	0.0486	1.61
P00167	CYB5	Cytochrome b5	4	0.56	0.0011	1.98
P00480	OTC	Ornithine carbamoyltransferase, mitochondrial	1	0.55	0.0349	2.36
Q13228	SBP1	Selenium-binding protein 1	2	0.55	0.0283	2.16
P07237	PDIA1	Protein disulfide-isomerase	14	0.45	0.0002	1.66
P05062	ALDOB	Fructose-bisphosphate aldolase B	10	0.38	0.0084	1.91
P07099	HYEP	Epoxide hydrolase 1	8	0.33	0.0313	1.82
P38646	GRP75	Stress-70 protein, mitochondrial	7	0.31	0.0449	1.66
P05091	ALDH2	Aldehyde dehydrogenase, mitochondrial	3	0.14	0.0161	2.18
Q9UNW1	MINP1	Multiple inositol polyphosphate phosphatase 1	1	0.01	0.0196	2.21
Q04917	1433F	14-3-3 protein eta	2	0.01	0.0173	2.36
Q6UX53	MET7B	Methyltransferase-like protein 7B	1	0.01	0.0184	2.18
O75608	LYPA1	Acyl-protein thioesterase 1	1	0.01	0.0184	2.35
Q16629	SFRS7	Splicing factor, arginine/serine-rich 7	1	0.01	0.0168	2.45
P10606	COX5B	Cytochrome c oxidase subunit 5B, mitochondrial	2	0.01	0.0182	2.28
P02786	TFR1	Transferrin receptor protein 1	1	0.01	0.0189	2.04
Q8N0X4	CLYBL	Citrate lyase subunit beta-like protein,mitochondrial	1	0.01	0.0176	2.44
O94905	ERLN2	Erlin-2	1	0.01	0.0191	2.33
P55263	ADK	Adenosine kinase	1	0.01	0.0199	2.27
Q9Y2Q3	GSTK1	Glutathione S-transferase kappa 1	1	0.01	0.0185	2.27
P62310	LSM3	U6 snRNA-associated Sm-like protein LSm3	2	0.01	0.0181	2.25
P04632	CPNS1	Calpain small subunit 1	1	0.01	0.0191	2.27
P84090	ERH	Enhancer of rudimentary homolog	2	0.01	0.0181	2.27
P48147	PPCE	Prolyl endopeptidase	1	0.01	0.0197	2.29
P05165	PCCA	Propionyl-CoA carboxylase alpha chain, mitochondrial	1	0.01	0.0195	2.29
P49588	SYAC	Alanyl-tRNA synthetase, cytoplasmic	2	0.01	0.0192	2.29
Q13561	DCTN2	Dynactin subunit 2	1	0.01	0.0192	2.29
Q07065	CKAP4	Cytoskeleton-associated protein 4	1	0.01	0.0192	2.29
Q9NVI7	ATD3A	ATPase family AAA domain-containing protein 3A	1	0.01	0.0191	2.29
Q16795	NDUA9	NADH dehydrogenase [ubiquinone] 1 alpha subcomplex subunit 9, mitochondrial	1	0.01	0.0190	2.29
Q00325	MPCP	Phosphate carrier protein, mitochondrial	1	0.01	0.0187	2.29
P27105	STOM	Erythrocyte band 7 integral membrane protein	1	0.01	0.0186	2.29
P30712	GSTT2	Glutathione S-transferase theta-2	1	0.01	0.0186	2.29
Q9Y262	IF3EI	Eukaryotic translation initiation factor 3 subunit E-interacting protein	1	0.01	0.0185	2.29
O95487	SC24B	Protein transport protein Sec24B	1	0.01	0.0184	2.29
P62263	RS14	40S ribosomal protein S14	1	0.01	0.0183	2.29
P13798	ACPH	Acylamino-acid-releasing enzyme	1	0.01	0.0181	2.29
O75964	ATP5L	ATP synthase subunit g, mitochondrial	1	0.01	0.0181	2.29
Q96I99	SUCB2	Succinyl-CoA ligase [GDP-forming] subunit beta, mitochondrial	1	0.01	0.0181	2.29
P50336	PPOX	Protoporphyrinogen oxidase	1	0.01	0.0180	2.29
P04196	HRG	Histidine-rich glycoprotein	1	0.01	0.0180	2.29
Q02318	CP27A	Cytochrome P450 27, mitochondrial	2	0.01	0.0179	2.29
P55157	MTP	Microsomal triglyceride transfer protein large subunit	1	0.01	0.0179	2.29
Q9BXK5	B2L13	Bcl-2-like 13 protein	1	0.01	0.0178	2.29
P51687	SUOX	Sulfite oxidase, mitochondrial	1	0.01	0.0177	2.29
Q9BVK6	TMED9	Transmembrane emp24 domain-containing protein 9	1	0.01	0.0177	2.29
Q96EY8	MMAB	Cob (I)yrinic acid a,c-diamide adenosyltransferase, mitochondrial	1	0.01	0.0177	2.29
P05093	CP17A	Cytochrome P450 17A1	1	0.01	0.0175	2.29
Q9Y5M8	SRPRB	Signal recognition particle receptor subunit beta	1	0.01	0.0173	2.29
P01011	AACT	Alpha-1-antichymotrypsin	1	0.01	0.0172	2.29
P02743	SAMP	Serum amyloid P-component	1	0.01	0.0166	2.29
Q02338	BDH	D-beta-hydroxybutyrate dehydrogenase, mitochondrial	1	0.01	0.0164	2.29

### Bioinformatics Analysis for the Sex-related Differential Proteins

**Figure 2:**
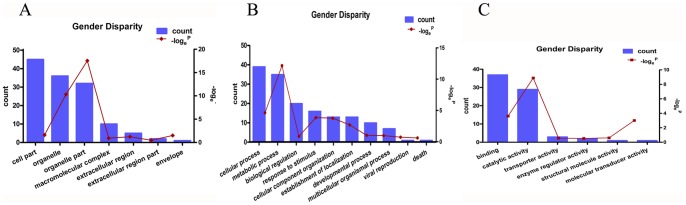
GO analysis for the differential proteins in HBV based HCC of sex-related disparity. A, Subcellular distributions of differential proteins; B, Biological process of differential proteins; C, Molecular function of differential proteins.

To clearly understand the role of the differential proteins in the sex-related disparity for HCC, we carried out Go (Gene Ontology) and Pathway (Reactome) analysis for the differential proteins using bioinformatics tools. The subcellular distributions for these differential proteins were mainly enriched in cytoplasm, organelles and nucleus (Figure 2A). Figure 2B showed the biological processes functional annotation of the identified proteins, these differential proteins were mainly involved in cell growth, metabolism, regulation and stress, such as, microsomal triglyceride transfer protein and stress-70 protein took part in the modification of protein and macromolecule biosynthetic process; Annexin A4 involved in signal transduction. Nearly 85% of the differential proteins were involved in biosynthesis and metabolic process, and 43.5% involved in signal transduction according to GO biological process analysis. Furthermore, molecular function analysis in the SWISS-PROT database indicated that the most common functional annotations of differential proteins were binding function. Besides, they also had catalytic activity, signal transducer activity and enzyme regulator activity (Figure 2C).

Further studies showed that all of the differential proteins were involved in 23 biological pathways in Reactome database. The metabolism of lipids and lipoproteins, pyruvate metabolism and citric acid cycle were proven to be associated with male hepatocarcinoma.

### Validation of Differential Proteins in Clinical Samples

**Figure 3:**
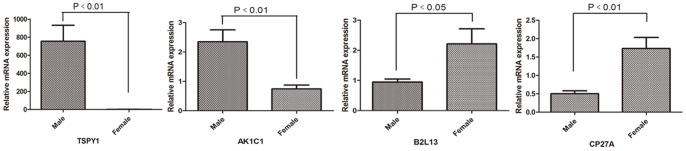
TSPY1, AK1C1, B2L13 and CP27A were detected in 38 male and 14 female HCC tissues by RT-PCR. TSPY1 and AK1C1 were increased in male HCC, B2L13 and CP27A were decreased in male HCC.

**Figure 4:**
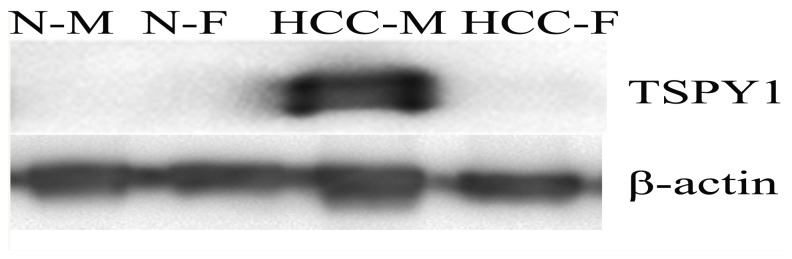
The result of western blot for TSPY1 protein in male and female HCC, and normal liver tissue (N-M: normal male liver tissue; N-F: normal female liver tissue; HCC-M: male HCC tissue; HCC-F: female HCC tissue).

In order to validate the iTRAQ results, four proteins out of the 73 differential proteins based sex-related disparity, i.e. TSPY1, AK1C1, B2L13 and CP27A were chosen for further validation in 38 male and 14 female HCC tissues. These proteins were selected according to the following criterion: (1) the big fold changes of differential expression between male and female HCC groups; (2) the biological behavior was strongly correlated with the occurrence and progression of cancer; (3) and its mechanism was unclear in HCC. The RT-PCR results indicated that the mRNA expression of those proteins were consistent with the iTRAQ results, TSPY1 and AK1C1 were increased, meanwhile B2L13 and CP27A were decreased in the male HCC tissues (Figure 3). Furthermore, after paid great attention, TSPY1 expression was much higher in male HCC group than that in female HCC group and normal group with significant difference analyzed by western blot, *p*<0.05(Figure 4).

TSPY1 is a member of the TSPY/SET/NAP1 superfamily mapped to the critical region harboring the gonadoblastoma locus which was the only oncogenic on the male-specific Y chromosome (GBY) [15], [16]. Therefore, TSPY1 was chosen for further study.

### TSPY1 Potentiates Cell Proliferation

**Figure 5:**
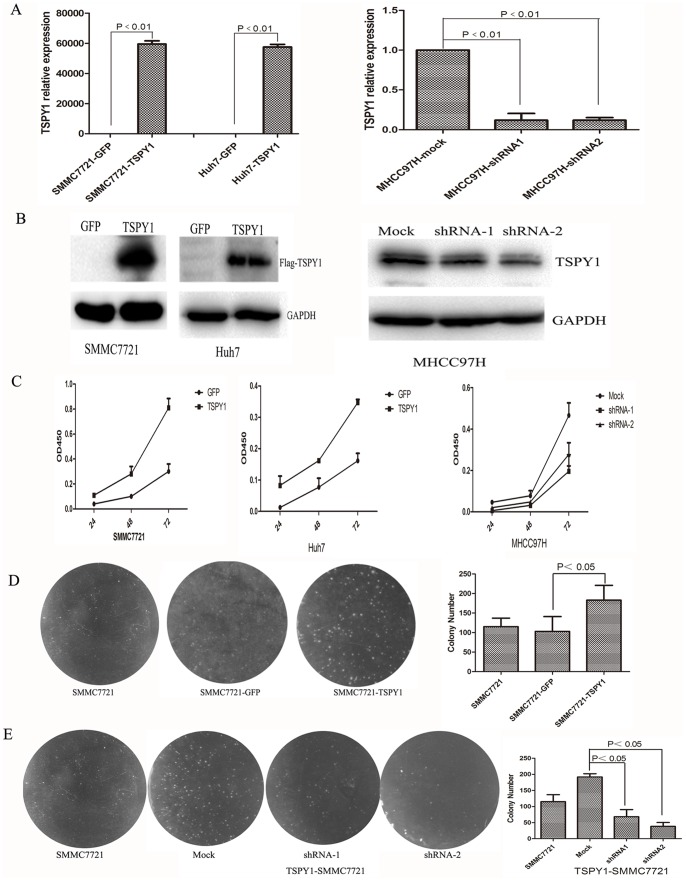
Function analysis of TSPY1 in HCC cells. (A and B), RT-PCR and western blot were used to detect TSPY1 expression in the TSPY1 over-expressing or knockdown HCC cells. C, CCK8 assay detected the cell proliferation after over-expressing or knockdown TSPY1. (D and E), Soft agar colonization of SMMC7721 cell over-expressing or knockdown TSPY1. All this data are from three independent experiments.

To determine whether TSPY1 plays a role in growth capability of HCC cells, we employed lentivirus encoding TSPY1 cDNA to over-express the expression of FLAG-tagged TSPY1 in SMMC7721 and Huh7 HCC cell lines. Also we used lentivirus-mediated shRNA to silence the expression of TSPY1 in MHCC97H cells. The expression of TSPY1 both in mRNA and protein levels were significantly increased after transfected with lentiviral encoding TSPY1 cDNA. And to the contrary, it was decreased in the MHCC97H cells silenced the expression of TSPY1 with shRNA (Figure 5A and 5B). CCK8 assay indicated that both in SMMC7721 and Huh7 cells over-expressed TSPY1 consistently presented higher proliferative activities than the control parent cells (Figure 5C). In the TSPY1 knockdown experiments, the cell proliferation were reduced obviously in the MHCC97H cells silenced the expression of TSPY1 with shRNA compared to the mock group (Figure 5C).

We also examined the rate of apoptosis under the circumstance of over-expressed or knockdown the expression of TSPY1 in HCC cells using flow cytometry analysis. The apoptosis rate in SMMC7721 cell over-expressed TSPY1(4.2±0.2%) was lower than the SMMC7721 cell transfected with the GFP-lentivirus (11.62±1.45%), *p*<0.05. The similar result was found in the comparison between Huh7 cell over-expressed TSPY1 (10.3±2%) and control cell (14.7±0.32%), but without significant difference (*p* = 0.19). While, in the TSPY1 knockdown experiments, the apoptosis rate of the shRNA-mediated suppression of TSPY1 in MHCC97-H cells were higher (14.7±1.87% for shRNA-1, 18.3±1.7% for shRNA-2) than the mock group (11.4±1.8%), and the *p* values were 0.2 and 0.06, respectively. These data implied that TSPY1 could promote cell proliferation through inhibiting apoptosis.

### Over-expression of TSPY1 Increases the Colony Formation in SMMC7721 Cell

Flag-TSPY1, GFP containing lentivirus transfected SMMC7721 cells were analyzed with soft agar colonization assay. It was found that TSPY1 over-expressed SMMC7721 cells acquired stronger ability in soft agar colonization than the cells transfected with GFP (Figure 5D). While we use the lentivirus-mediated shRNA to silence the expression of TSPY1 in the TSPY1 over-expressed SMMC7721 cell, the cell ability of soft agar colonization was significantly declined (Figure 5E). These findings suggest that ectopic expression of TSPY1 potentiates the efficiency of cell colony formation.

### TSPY1 Promotes Cell Invasion

**Figure 6:**
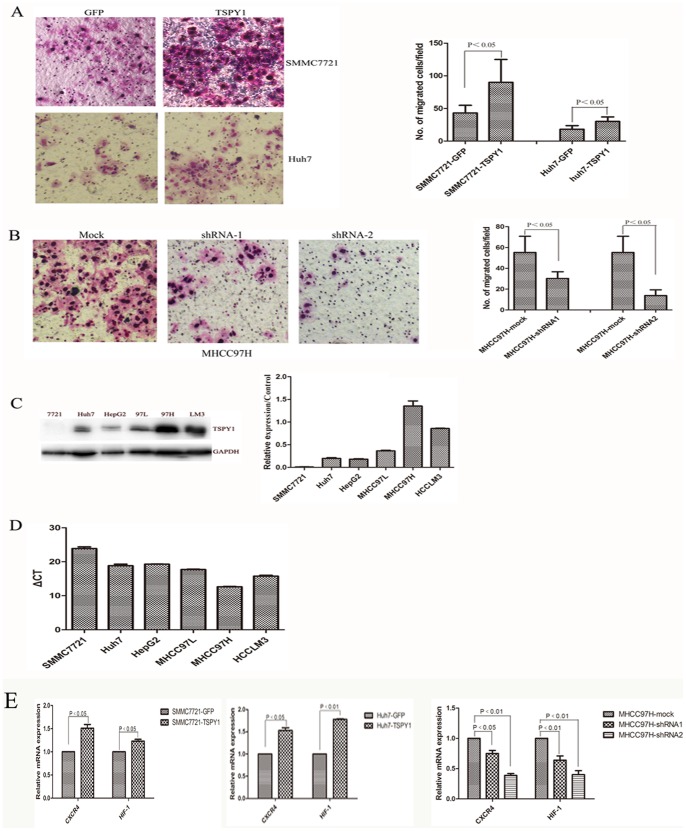
TSPY1 enhances invasion ablity of HCC cells. A, Over-expression of TSPY1 in SMMC7721 and Huh7 cells exhibited enhancing invasion ability compared to control cells (Data are shown as mean±SD, *p*<0.05, 100×). B, Silencing TSPY1 expression in MHCC97H cell inhibits cell invasion (Data are shown as mean±SD, *p*<0.05, 100×). C, Western blot was used to detect TSPY1 expression in HCC cell lines. D, RT-PCR was used to detect TSPY1 expression in HCC cell lines. E,Over-expressing TSPY1 increased the expression of CXCR4 and HIF-1 in SMMC7721 and Huh7, while suppressed CXCR4 and HIF-1 expression in the shRNA-TSPY1 MHCC97H cell. All this data are from three independent experiments.

To determine the effects of ectopic TSPY1 expression in cell invasion, Flag-TSPY1, shRNA transfected cells and the respective control cells were analyzed with the transwell invasion assay. TSPY1 over-expression in both SMMC7721 and Huh7 cells led to a marked increasing cell invasive ability (*p<*0.05, Figure 6A). While effective silencing of TSPY1 expression in MHCC97H cell significantly decreased the invasive ability compared to the mock group (*p*<0.05, Figure 6B). Analogously, the expression of TSPY1 in HCC cell lines was examined using western blot and RT-PCR. According to western blot analysis, the expression of TSPY1 was positively correlated with the metastatic potential of the HCC cell lines. TSPY1 protein levels in MHCC97H and HCCLM3 cells were significantly higher than those in MHCC97L, HepG2, SMMC7721 and Huh7 cells (Figure 6C). In concordance with the expression of protein, the mRNA level of TSPY1 also highly expressed in high metastatic MHCC97H and HCCLM3 cells, Figure 6D showed the value of △CT (△CT = CT_TSPY1_-CT_β-actin_) in HCC cell lines respectively. To determine the molecular basis of how TSPY1 enhanced invasive abilities of HCC cells, we next examined two invasion-related genes CXC chemokine receptor 4 (CXCR4) and hypoxia inducible factor-1 (HIF-1) that are known to play major role in tumor metastasis. Figure 6E showed the results of the RT-PCR from SMMC7721 and Huh7 over-expressing TSPY1, CXCR4 and HIF-1 were up-regulated about 1.5 fold. On the contrary, CXCR4 and HIF-1 were dramatically decreased in the knockdown study of MHCC97H cell (Figure 6E). These findings indicated that ectopic expression of TSPY1 was associated with HCC metastasis.

### TSPY1 may be Included in the Regulation of AR Expression Involved in Male HCC

**Figure 7:**
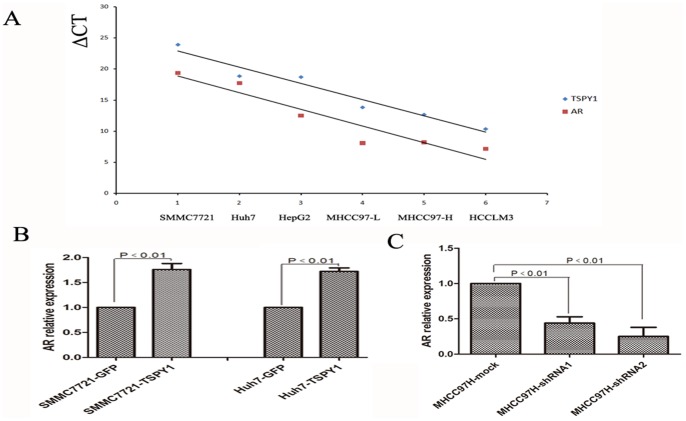
Over-expression of TSPY1 can up-regulate the expression of AR. A, TSPY1 and AR were similarly expressed among HCC cell lines analyzing by RT-PCR (△CT = CT_TSPY1/AR_−CT_β-actin_). (B and C), TSPY1 elevated expression of AR in SMMC7721 and Huh7 cells compared with the control cells, while AR was decreased in the shRNA-MHCC97H cells. All this data are from three independent experiments.

TSPY1 encoded on the mammalian Y chromosome was assumed to have male-specific functions. Like TSPY1, AR plays a pivotal role in male-specific biological events also [17]. Owing to TSPY1 was significantly up-regulated in male HCC; it may give rise to an interesting question whether there was a functional interplay between TSPY1 and AR in male HCC. To test our hypothesis, RT-PCR was used to examine the expression of TSPY1 and AR in HCC cell lines. The Pearson method was applied to assess the correlation between TSPY1 and AR. The results documented that TSPY1 and AR mRNA were coordinately expressed among HCC cell lines, and the correlation coefficient R was 0.84(*p<*0.01). TSPY1 and AR mRNA were co-expressed at a higher level in MHCC97H and HCCLM3 cells than in MHCC97L, HepG2, SMMC7721 and Huh7 cells (Figure 7A). To investigate the expression of TSPY1 and AR in HCC tissue, RT-PCR was used to detect the expression of AR in male HCC tissue dots. The result of western blot from male HCC tissue suggested that TSPY1 protein had increased along with AR expression (data not show).

The mRNA expression of AR in HCC cells under the conditions of TSPY1 over-expression and down-regulation was also determined. It was found that increased expression of TSPY1 in SMMC7721 and Huh7 cells caused an elevated expression of AR by more than 1.5-fold compared to the control cells (Figure 7B). While knockdown of TSPY1 resulted in a substantial decreased expression of AR in MHCC97H cells (Figure 7C). According to the previous study that AR could promote HCC progression, we can speculate that TSPY1 may be included in the regulation of AR expression involved in male HCC.

## Discussion

HCC is a male-predominant cancer associated with chronic viral hepatitis. In our study, quantitative proteomics technology was used to screen the differential proteins between male and female HCC tissue. All specimens excluded the recurrence and chemotherapy cases were confirmed as primary HCC without metastasis by clinical pathology. This could reduce the heterogeneity among tissues. Finally 109 differential proteins were identified through iTRAQ-2DLC-ESI-MS/MS analysis between normal and HCC tissues. Interestingly, some of them were definitely expressed both in normal and HCC tissues. In case of cytochrome b5(CYB5), it expressed at a higher level in normal male tissue than in normal female, but its expression was lower in male HCC than in female HCC tissue. Cytochrome P450 can convert testosterone to eight metabolites inactivating testosterone in the liver [18]. The decreased expression of CYB5 could increase the amount of testosterone which conducive to male HCC via androgen pathway. HBV was the leading cause of human HCC. The ability of stress resistance for host hepatocytes was reduced during the progress of HBV reproduction and leading oxidative damage of hepatocytes. In contrast to the normal group, the oxidative stress related proteins Cytochrome P450 17A1(CYP27A1), Fructose-bisphosphate aldolase B (ALDOB), Stress-70 protein (GRP75), Ornithine carbamoyltransferase (OTC) and so on were decreased in the HCC group. It is noteworthy that a large number of antioxidant proteasome were down-regulated in the male HCC tissue. So the oxidative damage caused by HBV of male is more serious than that of female, it may be the potential mechanisms leading the fast progress of HCC in male than in female.

Thirteen of the differential proteins we screened have been identified in previous study [19]. For example, heat shock protein beta-1(HSPB1) was found as increasing biomarker for HCC. TSPY1 was chosen for further study since (1) TSPY1was only found in male HCC group with a significant difference. (2) It was considered as a candidate oncogene for gonadoblastoma [20] and (3) the exact mechanisms of its postulated oncogenic are still unclear. TSPY1 is a member of the TSPY superfamily which located on the pericentromeric region of the short arm on the Y chromosome [21]. TSPY is normally expressed in testis predominantly in spermatogonia and serve vital functions in male germ cell development and spermatogenesis [22]. The abnormal expression of TSPY were observed in early and late stages of gonadoblastoma, testicular carcinoma in situ [23], prostate cancer [24] and melanoma [25]. In our study, the male-specific TSPY1 was significantly elevated with the comparison of male HCC tissue to female HCC tissue. This result was verified by RT-PCR and western blot using male and female clinic tissues. In concordance with previous study, TSPY was up-regulated in HCC patients suggested that it was a novel cancer testis antigen and a potential candidate in vaccine strategy for immunotherapy in HCC patients [26].

A series of experiments were performed to determine the role of TSPY1 in HCC cells. We found that TSPY1 could potentiate HCC cells proliferation and inhibit apoptosis. TSPY can bind cyclin B at its SET/NAP domains, enhance cyclin B-CDK1 kinase activity, and promote cell proliferation via propelling a rapid G2/M transition in the cell cycle [23], [27]. Shane found that over-expression of TSPY promoted cell proliferation in HeLa and NIH3T3 cells and the expression of TSPY can affect numerous cell cycle and apoptosis gene analyzed by microarray analysis [28]. TSPY play a catalytic role in the development of many cancers. Tatsuo suggested that TSPY could interact with translation elongation factor eEF1A, via enhancing protein synthesis and gene transcription to exert its oncogenic function [29]. In our study, we also confirmed that ectopic expression of TSPY1 in HCC SMMC7721 cell increased the colony formation efficiency. This result was consistent with the previous study which indicated that TSPY formed higher numbers of colonies in vitro and enhanced tumor growth in vivo [28]. Lauren demonstrated that TSPY1 presented in most gonadoblastomas using interphase fluorescent in situ hybridization assay [20]. All of our data taken together supported the notion that TSPY1 was a growth-promoting gene and provided a new insight of abundant TSPY1 expression in male HCC tissues. Furthermore, we also found that recombinant TSPY1 significantly increased invasive ability of HCC cells. This is the first study to determine the effects of TSPY1 in cell invasion. Meanwhile, TSPY1 was also significantly high expressed in high metastatic MHCC97H and HCCLM3 cells which confirmed our results plenty. However, mechanisms of TSPY1 how to influence tumor metastasis are still unclear. CXCR4 and HIF-1 were significantly up-regulated in over-expressing TSPY1 SMMC7721 and Huh7 cells, while down-regulated in the TSPY1 knockdown MHCC97H cell. Previous study indicated that CXCR4 were highly expressed in HCC, and its ligand chemokine (C-X-C motif) ligand 12 (CXCL12) CXCL12 can stimulate human hepatoma cell growth, migration and invasion [30]. The level of CXCR4 also associated with lymph node metastasis of HCC, it was considered as an independent prognostic factor for HCC with lymph node metastasis [31]. Esther found that over-activation of the TGF-β pathway via increasing expression of CXCR4 conferred HCC cells migratory properties [32]. HIF-1 plays a key role in tumor angiogenesis because of activation human VEGF genes. Its subunit (HIF-1α) over-expressed in HCC was significantly associated with tumor angiogenesis, invasion and metastasis and poor prognosis [33]. All these results indicated that TSPY1 played a critical role in HCC metastasis via interacting with invasion-related factors.

TSPY1 and AR were both encoded by Y chromosome gene inextricably linked with male. The liver is the target organ of androgen action, as AR can enhance HBV transcription promoting hepatocarcinogenesis and TSPY1 also highly expressed in male HCC tissue, cross-talk between AR and TSPY1 is conceivable. In the present study, TSPY1 and AR had a dramatic positive correlation in HCC cell lines as well as in male HCC tissue. TSPY1 and AR were expressed at a high level in high metastatic HCC cells and a low level in low or no metastatic HCC cells. In HBV based male HCC tissue, TSPY1 was increased with the AR expression. To further validate the correlation between TSPY1 and AR, we used full length TSPY1 cDNA and shRNA-TSPY1 virus transected HCC cells. The results showed that over-expression of TSPY1 significantly increased the expression of AR, while knockdown of TSPY1 resulted in a substantial decreasing AR level. These results indicated that TSPY1 may be included in the regulation of AR expression involved in male HCC. However, the AR coregulatory function of TSPY1 in the male HCC remains unclear. Future work will aim to clarify the mechanism of TSPY1 participate in the regulation of AR expression involved in male HCC progress.

In summary, TSPY1 was identified and significantly increased in male HCC tissues. We demonstrate that TSPY1 can potentiate the ability of cell proliferation, colony formation and invasion. Our data also indicate that TSPY1 may be involved in male HCC progress via participating in the regulation of AR expression. These findings will present new insights into mechanism of male HCC and provide a potential therapeutic target.
